# Murine leukocyte dysfunction in response to a non-lethal flame burn

**DOI:** 10.1128/iai.00604-25

**Published:** 2025-12-23

**Authors:** Adrienne R. Kambouris, Jerod A. Brammer, Gideon Wolf, Amit Kumar, Alan S. Cross

**Affiliations:** 1 Center for Vaccine Development and Global Health, University of Maryland School of Medicine 12264, Baltimore, Maryland, USA; 2 US Army Institute of Surgical Research 110230 , Joint Base San Antonio Fort Sam Houston, Texas, USA; 3 Department of Microbiology and Immunology, University of Maryland School of Medicine 12264, Baltimore, Maryland, USA; St Jude Children's Research Hospital, Memphis, Tennessee, USA

**Keywords:** DAMP, HMGB1, leukocyte, *Pseudomonas aeruginosa*, burn injury

## Abstract

In a non-lethal, 10% total body surface area, full-thickness flame mouse model, infections with *Pseudomonas aeruginosa* (PA) increased mortality post-burn, suggesting an impaired host immune response. The presence of a seroma beneath the burn wound sequesters CD45^+^ cells. Furthermore, in the case of burn and infection, PA was found to be in proximity to these cells but was not phagocytosed, suggesting leukocyte dysfunction. In this study, leukocytes isolated from the circulation and seroma of burned mice had a decreased ability to kill PA compared to the circulating leukocytes of Sham mice. Both Sham and burned mouse leukocytes lost the ability to kill when incubated *in vitro* with seroma fluid. Leukocytes from the seroma had a decreased ability to produce reactive oxygen species (ROS) following stimulation when compared to leukocytes isolated from the circulation of the same burned mice. Sham leukocytes incubated with sera from burned mice and burned and infected mice, but not with sera from Sham mice, significantly produce ROS at rest, which may be correlated with the pro-inflammatory danger-associated molecular pattern (DAMP) HMGB1 in the sera of burned mice. These data suggest that a non-lethal burn can prematurely activate leukocytes while in circulation, reducing their functionality at the infected burn site, and that leukocytes at the burn site (seroma) also have impaired function. We conclude that an otherwise non-lethal burn prematurely activates circulating leukocytes and that the seroma environment further inhibits the leukocytes that arrive at the burn site. This results in an impaired immune response and the development of lethal sepsis.

## INTRODUCTION

Burns are a leading cause of mortality worldwide, causing over 100,000 deaths annually (1). Patients experiencing burn wounds are evaluated based on the depth and total body surface area (TBSA) involved in the injury. There is a positive correlation between the extent of TBSA and risk for mortality (2–5). We previously reported that an otherwise non-lethal burn injury (10% TBSA) may become lethal if there is a superimposed *Pseudomonas aeruginosa* (PA) infection, one of the most common causes of death in burn patients (4, 6, 7). While PA infections are less common in hosts with an intact immune system, PA can establish infection in hosts with an impaired leukocyte response (8–11), particularly in patients with chemotherapy-induced neutropenia or following burn injury (4, 6, 12–16).

In a previous study, we observed that the lethal dose of bacteria was reduced from 10^7^ CFU in the absence of the burn to approximately 10^3^ CFU with the burn (17). Furthermore, while mice immediately infected with PA post-burn experienced 100% mortality within 36 hours (17), mice infected with the same PA challenge dose at 72 hours post-burn had restored survival. This suggests that the burn injury transiently alters immune defenses, making the host more susceptible to infection. There was also a collection of gelatinous fluid between the skin and the dorsal fascia at the burn site (18). This seroma appeared at approximately 3 hours post-burn and began to recede at approximately 18 hours. Seroma formation was accompanied by an influx of CD45+ cells. The leukocytes sequestered in the seroma resulted in a functional peripheral leukopenia, a major risk factor for *Pseudomonas* dissemination. Within the seroma, a large number of bacteria were in proximity, but not within, the leukocytes, suggesting that, in addition to the peripheral leukopenia, leukocyte dysfunction at the burn site might also contribute to the increased susceptibility and lethality of bacterial infection during burn injury. Given the critical role of neutrophils in the resolution of *Pseudomonas* infection, in the current study, we examine the leukocyte population in the seroma and the potential role of burn-induced neutrophil dysfunction in the altered immune response (19–21).

Burn wounds release from cells danger-associated molecular patterns (DAMPs), such as mitochondrial DNA (mtDNA), high mobility group box 1 (HMGB1), and S100A, into circulation (22–24). Increased levels of circulating DAMPs post-burn serve as a predictor of mortality (22, 25). HMGB1 is released into the circulation post-burn at levels that bind to MD-2 in the TLR4 complex and result in a robust pro-inflammatory response (17). We observed that, while HMGB1 was released into circulation immediately post-burn (approximately 50 ng/mL, returning to baseline after 3 hours), a 10-fold increase from baseline following a burn and infection was observed, which steadily increased until death (17). Survival was restored when burned and infected mice were administered P5779, which competes with HMGB1 for its binding site on MD-2 (17).

These observations lead to the hypothesis that leukocytes may be experiencing premature activation in circulation and arrive at the burn site “exhausted” or having utilized their prepared functions. Combined with the ability of the seroma fluid to support the continued growth of PA, these factors may contribute to the observed increased mortality in our model. To assess the function of leukocytes from the circulation and from the seroma of burned mice, we compared these leukocytes to those isolated from Sham mice. While the ability of leukocytes in the circulation and seroma of burned mice to kill PA was similar, their bactericidal activities were significantly decreased compared to those of leukocytes from naïve mice. Additionally, we observed that naïve leukocytes resuspended in seroma fluid had a decreased ability to kill, either due to inhibition by the seroma fluid or the continued growth of PA. There was a decrease in ROS production in leukocytes isolated from the seroma of burned mice when compared to leukocytes in the circulation of the same mice. Leukocytes from naïve mice (i.e., mice that did not have the hair clipped, receive anesthesia, or resuscitation fluids, as occurred with Sham mice) incubated with sera from burned and infected mice with quantified HMGB1 produced ROS in the absence of stimulation. Together, a non-lethal burn impacted leukocyte function not only at the burn site but also in the circulation.

## MATERIALS AND METHODS

### Bacterial preparation

The *Pseudomonas aeruginosa* M2 isolate (IATS O5) was kindly provided by Dr. Alan Holder, formerly of the Shriners Burn Institute at the University of Cincinnati. *Pseudomonas* glycerol stocks were isolated on tryptic soy agar (TSA) via streaking (Sigma-Aldrich, St. Louis, MO) plates and incubated at 37°C for 18 hours. A single colony was transferred to 3.0 mL of Hy-Soy Broth, containing 0.5% sodium chloride (American Bio, Canton, MA), 0.5% HY-Yeast (Kerry Bio-Science, Norwich, NY), and 0.25% animal-free soytone (Teknova, Hollister, CA) and grown in a shaking incubator at 225 rpm at 37°C to the stationary phase. Two hundred forty microliters of overnight inoculum was added to 12 mL of Hy-Soy broth and grown in a shaking incubator until log phase, OD_600_ of 0.2–0.3 at 37°C. The bacteria were pelleted, washed twice with sterile phosphate-buffered saline (PBS), and resuspended in PBS to the desired concentration.

### Burn and infection procedure

With the approval of the University of Maryland, Baltimore, IACUC Protocol 0322001, the burn procedure was performed using the Stieritz and Holder method (12) as previously described (17, 18, 26). Briefly, female Crl:CD1 mice (Charles River Laboratories, MA) between 8 and 10 weeks old had their dorsal hair clipped 24 hours before the burn procedure. Twenty-four hours later, mice were administered 5% isoflurane for 7 minutes; a toe pinch was performed before each burn to ensure that the mice were successfully anesthetized. Mice were placed on their abdomen in a chemical fume hood, and a flame-resistant polymer template delineating roughly 10% TBSA (2.5 cm × 4.0 cm) was pressed down on the clipped area of the back. With a glass dropper, 500 µL of 100% ethanol was deposited onto the exposed area within the template. The ethanol was ignited using a lighter; a timer was used to ensure the burn lasted exactly 10 s. The flame was extinguished by breath. Immediately post-burn, mice were administered 500 µL of Ringer’s solution i.p. for fluid resuscitation. Mice were placed in their original cages for monitoring and anesthesia recovery. Sham mice received the same treatment as experimental mice (i.e*.,* clip/anesthesia/resuscitation) except for burn and infection. In the instance of infection, mice were infected with 100 µL of 1 × 10^6^ CFU/mL of PA subcutaneously at the burn site directly after the burn.

### Flow cytometry

Blood and seroma samples were taken from Sham, burn, and burn and infected mice at 6, 12, and 18 hours post-burn. Three mice were used in each condition. If a sample could not be collected and only two mice are included, it is indicated in the associated figure. Blood cell suspension was prepared using ACK lysis. Seroma samples were digested using 2 mL of a collagenase solution (2.5 g/mL collagenase D and 200 U/mL Dnase 1) for 2 hours at 37°C, then pressed through a 70 µM cell strainer. All cells were pelleted at 1,200 rpm for 7 minutes at 4°C and resuspended in an Fc-block cocktail of mouse, rat, and hamster IgG, and anti-CD16/32 rat anti-mouse IgG clone 2.4G2. The cells were incubated with the following primary antibodies for 15 minutes at 4°C (Table 1).

**TABLE 1:** Flow cytometry antibody panel used to identify cells in the blood and seroma of mice^*a*^

Marker	Color	Cell stain (μL)	Total volume (μL)	Clone
CD44	BV421	0.3	19.5	IM7
CD45R	PacBlue	0.3	19.5	RA3-6B2
CD11b	BV480	1	65	M1/70
CD8a	BV510	1	65	53-6.7
CD45	BV570	0.5	32.5	30-F11
PDCA-1	BV605	0.5	32.5	927
CD27	BV650	0.5	32.5	LG.3A10
CD11c	BV711	0.5	32.5	N418
CD4	BV750	0.5	32.5	GK1.5
Ly6C	BV785	0.05	3.25	HK1.4
CD3	Spk550	1	65	17A2
F4/80	PE-Dazzle 594	1	65	BM8
CD49b	PE-Cy5	0.625	40.625	DX5
Ly6G	PercP	1.5	97.5	1A8
CD69	PercP-Cy5.5	1.5	97.5	H1.2F3
CD1d	PE-Cy7	1	65	1B1
CD62L	Alexa 700	1	65	MEL-14
CD14	APC-Fire 750	1	65	Sa14-2

^*a*^ All cell samples were stained using the markers in this panel. The fluorochrome color, staining volume, and clone information for each marker are indicated. Each antibody was titrated for optimal signal-to-noise ratio in multicolor staining.

Following incubation, the cells were washed, centrifuged, and resuspended in 500 µL of 1× FACS buffer. Cells were gated with Zombie-NIR for living cells. They were then gated to isolate neutrophils, selecting CD45^+^ and CD3^−^, then CD45^+^ and B220^−^ to eliminate T and B cells. Finally, cells that were CD11b^+^ and Ly6G^hi^ were analyzed as neutrophils. Flow cytometry was performed using the Cytek Aurora flow cytometer (Cytek Biosciences, Fremont, CA), and analysis was performed using FlowJo (BD Biosciences).

### Neutrophil isolation

After administering isoflurane at the previously described dose, mice were placed on their backs and their thoraces were sterilized with 70% ethanol. Blood samples were collected via cardiac puncture with a 1 mL syringe and 25 g needle. Whole blood was collected and stored in EDTA tubes to prevent clotting. Red blood cells were lysed using ACK Lysis Buffer (27), according to the manufacturer’s protocol. For seroma isolation, the collected seroma fluid was incubated with 2 mL of 2.5 g/mL of collagenase in PBS for 2 hours at 37°C, and the samples were passed through a 70 µm cell strainer. After washing, the resuspended pellets were then overlayed on a Histopaque-based density gradient (1.119 g/mL and 1.077 g/mL; Sigma-Aldrich, St. Louis, MO) and centrifuged at 872 × *g* for 30 minutes (28). The cell layer was carefully removed and washed in HBSS^−^ and spun at 1,400 rpm for 7 minutes with no brake. Live cells were counted using trypan blue dye exclusion (>90% viable cells) and immediately used in functional assays. Each experiment was conducted with pooled blood from 5 mice. Isolation of neutrophils was verified with flow cytometry with 80% viability.

### HMGB1 quantification

Blood was collected at 18 hours post-burn and infection as previously described and then centrifuged in 1 mL serum gel tubes (Sarstedt, Newton, NC) at 12,000 × *g* for 10 minutes. 100 µL of each sample was aliquoted for quantification, and the remaining sample was snap-frozen using liquid nitrogen. Samples were quantified using a Mouse HMGB1 ELISA kit (Novus Biologicals, CO), and the assay was completed according to the manufacturer’s instructions.

### Bacterial killing assay

Isolated leukocytes diluted to 1 × 10^6^ were incubated in 96-well plates with log-phase PA at a 1:1 ratio. In seroma supplement experiments, leukocytes were resuspended in 250 µL of seroma in the 25% condition and 750 µL in the 75% condition, then diluted to 1 mL total with HBSS^+^. Immediately after plating, a sample was taken for serial dilution to quantify the starting inoculum. The plates were placed on a shaker at 150 rpm in a 37 °C incubator for 2 hours. A second sample was taken for serial dilution. The dilutions were grown on TSA plates overnight at 31°C and counted. Data were visualized using Prism, and statistics were analyzed by one-way ANOVA with multiple comparisons.

### Cytochrome C reduction assay

Leukocyte samples were diluted to 1 × 10^6^ cells/mL in each trial. In a 96-well plate, cells (15 µL) were incubated with cytochrome C (1.5 µL) and HBSS^+^ (5 µL and diluent to 31.5 µL), and SOD inhibitor (SODi) (5 µL), 10 µg/mL phorbol myristate acetate (PMA) (5 µL), or both. For 20 minutes, the plate was shaken at 150 rpm in a 37°C incubator. Colorimetric readings were taken at 550 nm using a plate reader. In Sham leukocyte experiments, 5 µL of serum was added in place of PMA. Data were visualized using Prism and analyzed with the REML mixed model with multiple comparisons.

## RESULTS

### Recruited cells to the seroma were previously activated

**Fig 1:**
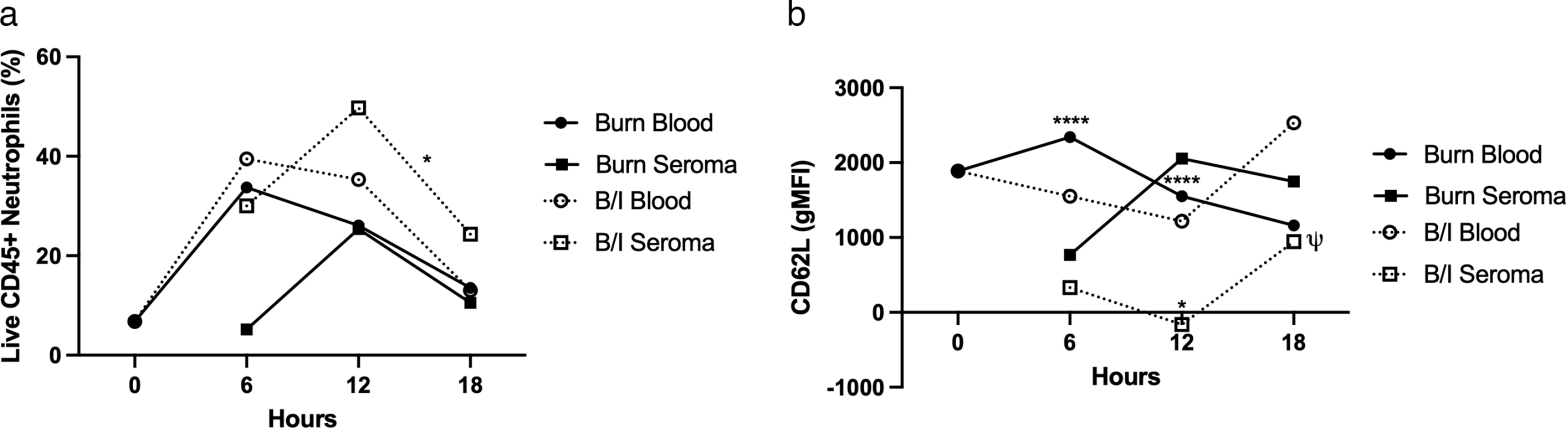
Three mice were either burned or burned and infected (B/I), and samples were collected at the time points indicated. After sample preparation for flow cytometry, live CD45^+^ cells were isolated through gating as previously described. This population was then characterized in each tissue sample site. ψ indicates that samples consist of two mice instead of three. The data were analyzed using two-way ANOVA with multiple comparisons. The measurements from the blood of Sham mice were used as the 0 hour timepoint to show changes from baseline. (**a**) Percent of CD11b^+^ Ly6G^hi^ cells in each time, condition, and site. B/I seroma 12 hours vs B/I seroma 18 hours *P* = 0.0263 (**b**) gMFI of CD62L in each tissue. Burn blood 6 hours vs burn seroma 6 hours *P* < 0.0001; Burn blood 12 hours vs burn seroma 12 hours *P* < 0.0001; burn seroma 12 hours vs B/I seroma 12 hours *P* = 0.0115.

Using flow cytometry, we analyzed cells from the blood and seroma of burned and infected (B/I) mice (Table 1). Among CD45^+^ cells, the seroma contained a diverse population including neutrophils (Fig. 1a), macrophages (CD14^+^, F4/80^+^; comprising 1.7%–51.6% of CD45^+^ cells depending on the condition and time point), and a small number of γδ T cells (CD3^+^, CD4^−^, CD8^−^).

Neutrophil dynamics showed a temporal pattern in the population of live CD45^+^, CD11b^+^, and Ly6G^hi^ cells (Fig. 1a). In blood, using the percentage of polymorphonuclear neutrophils (PMNs) in Sham mice as the baseline (time 0), PMNs peaked at 6 hours post-burn (mean 33.7%) and declined over time. In contrast, in the seroma, PMN levels peaked later, at 12 hours post-burn (25.3%). Under the B/I condition, PMN recruitment to the seroma increased further, peaking at 49.7% (Fig. 1a).

To assess PMN activation, we examined CD62L expression. CD62L is a surface marker shed from PMNs upon activation. In the seroma under B/I conditions, although PMN numbers increased, CD62L expression decreased at corresponding time points (Fig. 1b). In the blood of Burn-only mice, unactivated PMNs (CD62L^+^) increased at 6 hours but declined thereafter. In contrast, CD62L levels in the B/I blood samples steadily dropped, followed by a sharp increase at 18 hours post-injury and infection.

In the burn condition seroma, CD62L intensity peaked at 12 hours, mirroring the PMN percentage trend. However, in the B/I seroma, CD62L levels dropped sharply at the same time point, despite a higher proportion of live PMNs. Together, these blood and seroma data suggest that the neutrophils recruited in the B/I condition were previously activated.

### Burn alone is sufficient to impact leukocyte killing

**Fig 2:**
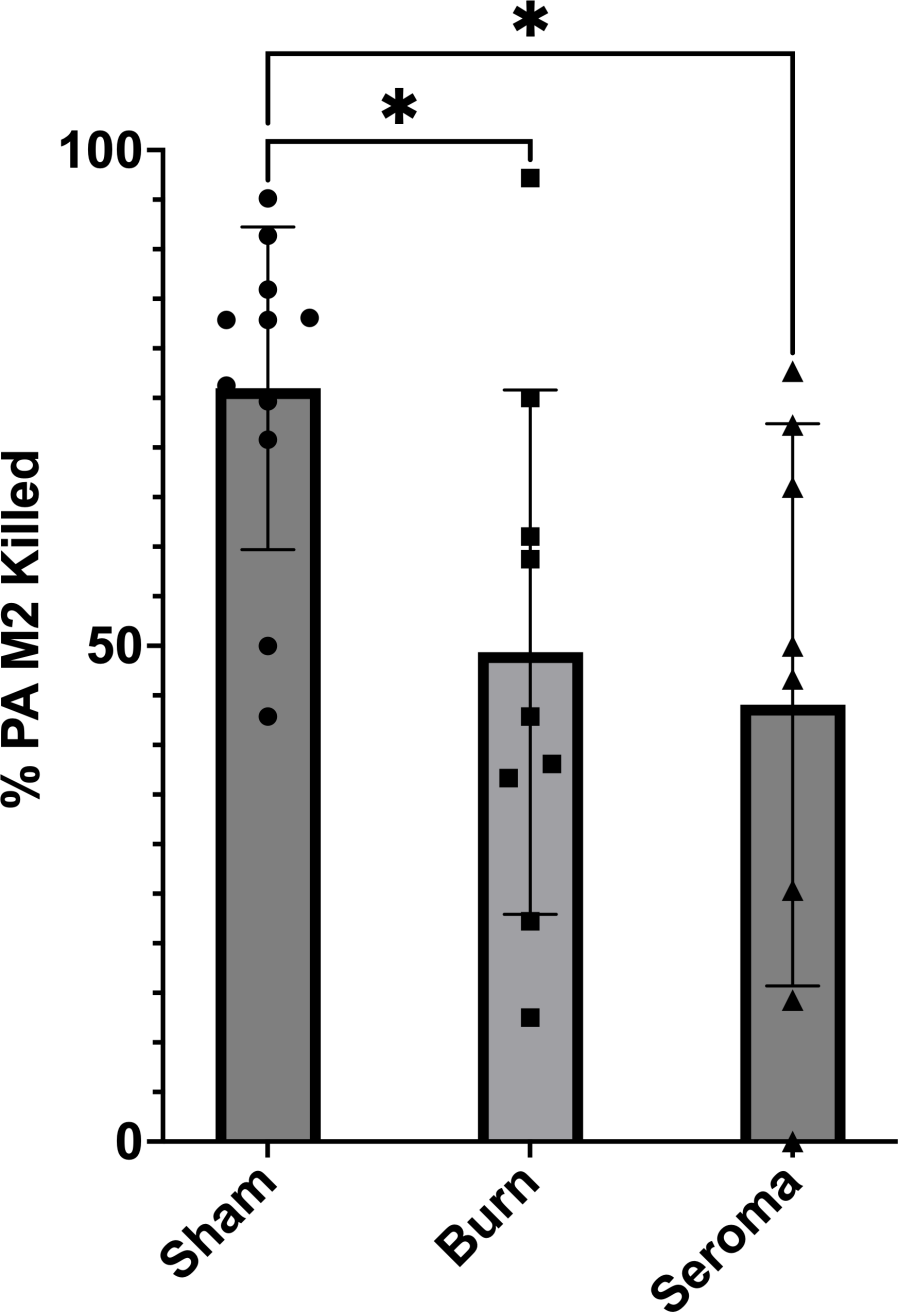
Phagocytic activity of neutrophils from the circulation of Sham mice, and from the circulation and seroma of burned mice resuspended in HBSS+. Neutrophils pooled from five mice (the circulation of Sham mice, the circulation of burned mice [Burn], and from the seroma from the same burned mice [Seroma]) were incubated with PA M2 in HBSS^+^ at 37°C for 2 hours. Samples were taken at time 0 and at the end of the 2 hours. Serial dilutions of the 10 µL samples were plated on TSA and incubated overnight. The CFUs were counted and calculated to adjust for dilution. The data charted is 1−(Time 2 hours/Time 0 hour). Bar height is the mean, and error bars indicate standard deviation. *P < 0.05.

To determine whether burn injury impacts the ability of leukocytes to kill bacteria, we incubated PA with leukocytes isolated from the circulation and seroma of the same burned mice. These findings were compared to the bactericidal activity of the circulating leukocytes of Sham mice when incubated with PA at an MOI of 1 for 2 hours at 37°C. Surprisingly, the leukocytes in the circulation were impacted by the burn alone, having a significant decrease in bacterial killing when compared to leukocytes in the circulation of Sham mice (Fig. 2). These data suggest that substances in the circulation from a non-lethal burn are sufficient to impact leukocyte function even before they reach the seroma environment.

### In seroma fluid, leukocytes lose the ability to kill PA

**Fig 3:**
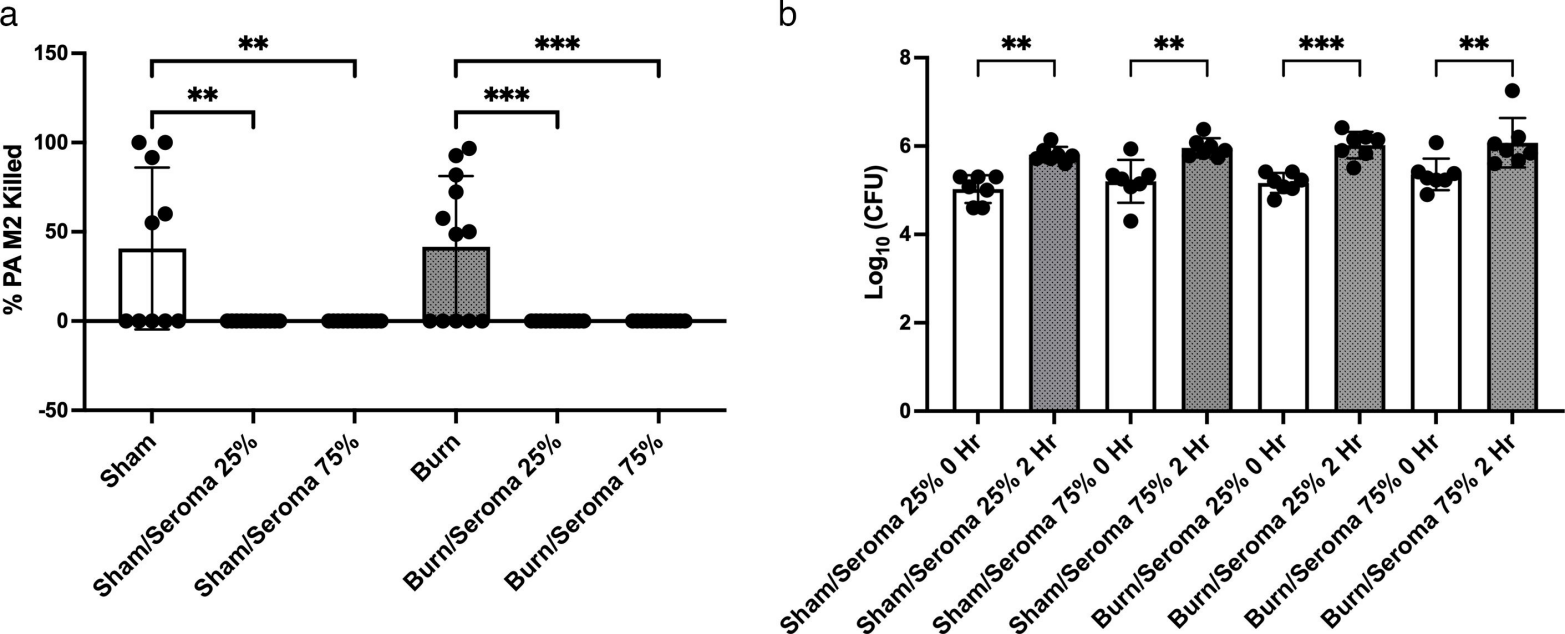
Phagocytic activity of neutrophils from the peripheral blood of Sham and from burned mice resuspended in seroma fluid. Neutrophils were incubated with PA M2 in HBSS^+^ and seroma (+, 25%; ++, 75%) at 37°C for 2 hours. Samples were taken at time 0 and at the end of the 2 hours. Serial dilutions of the 10 µL samples were plated on TSA and incubated overnight. The CFUs were counted and calculated to adjust for dilution. The data charted is 1 − (Time 2 hours/Time 0 hour). (**a**) Percent of PA killed after 2 hours. (**b**) Actual CFU counts from all samples after 2 hours. **P < 0.01, ***P < 0.001.

To assess the role of the seroma environment in leukocyte bactericidal activity, seroma fluid was collected from burned (but uninfected) mice and used to resuspend isolated leukocytes from the circulation of Sham and burned mice. The cells were resuspended in HBSS^+^ alone or HBSS^+^ enriched with either 25% or 75% seroma fluid. After 2 hours of incubation, leukocytes from the circulation of Sham and burned mice could kill PA; however, in the HBSS/seroma-enriched treatments, leukocytes lost the ability to kill bacteria (Fig. 3a). In fact, there were significantly more CFUs present after 2 hours in all samples that were incubated in seroma fluid (Fig. 3b), confirming previous work showing that the seroma environment supports PA growth (18). It is unclear whether the seroma fluid directly inhibited killing by leukocytes or if the PA was simply able to continue to grow in the seroma environment.

### Leukocytes in the seroma have a decreased ability to generate ROS

**Fig 4:**
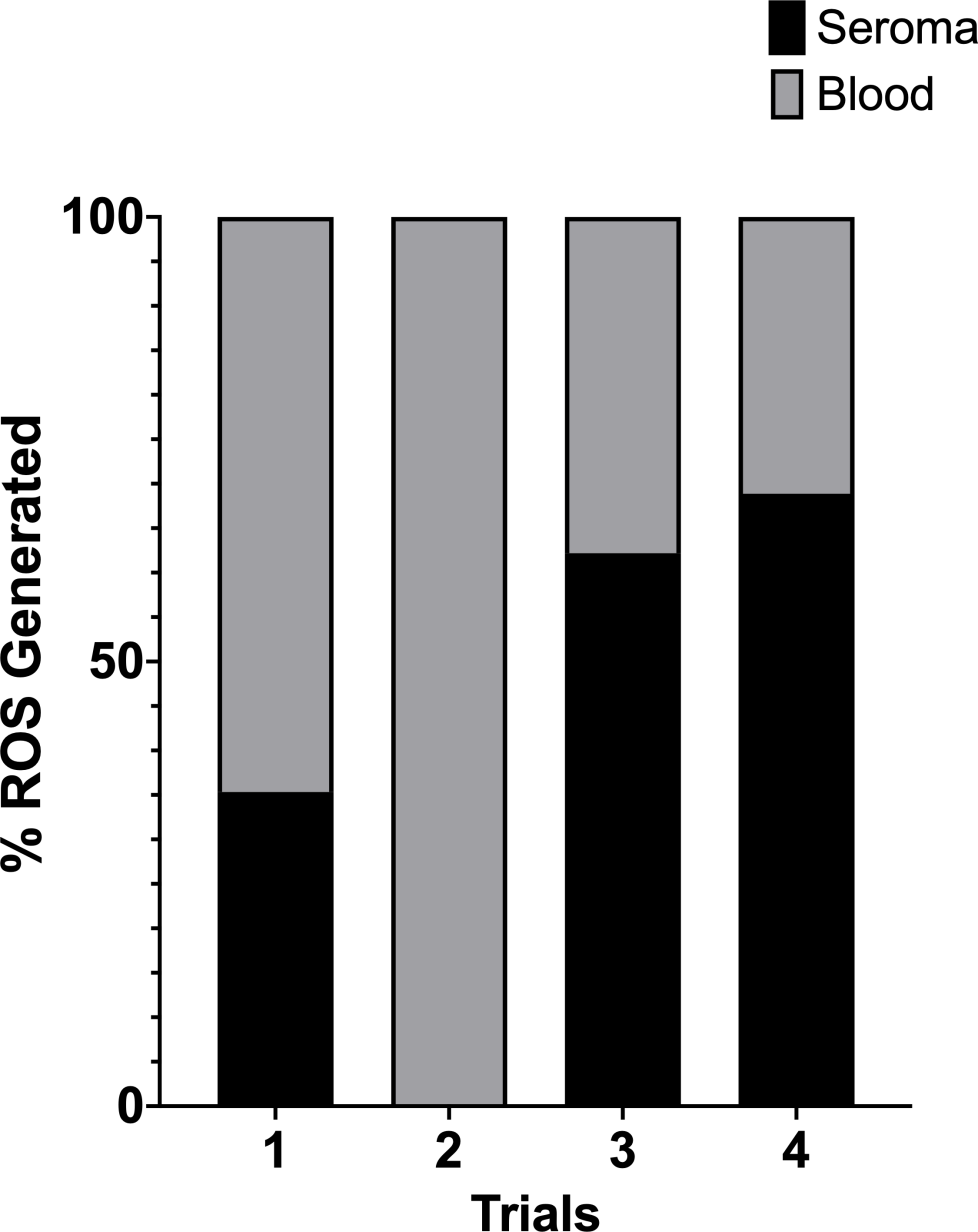
ROS production in seroma-isolated neutrophils in relation to those isolated from circulation. Each trial consists of the pooled neutrophils of 5 mice, comparing the activity of neutrophils collected from the seroma and the blood. Both blood and seroma were collected from five mice at 18 hours post-burn. The production of neutrophils isolated from blood was normalized to 100%, and the activity of the seroma neutrophils is shown as a fraction. Reduction was measured through colorimetry at 550 nm.

To address this possibility, we utilized a cytochrome C assay to measure reactive oxygen species (ROS) production. Leukocytes release ROS into the phagosome, which is toxic to pathogens by damaging membranes, proteins, and DNA. We compared pooled leukocytes from the circulation of five burned mice to leukocytes isolated from the seroma of the burn site of the same mice. The leukocytes were resuspended in HBSS^+^ stimulated with PMA, then incubated for 20 minutes at 37°C. We set the ROS production of the circulating leukocytes as 100% and expressed the production of the leukocytes in the seroma as a percentage of the ROS production of the circulating leukocytes (Fig. 4). In each instance, the seroma leukocytes had a decreased ability to produce ROS.

### Sham leukocytes incubated in sera with HMGB1 reduce cytochrome C in the absence of exogenous stimulation

**Fig 5:**
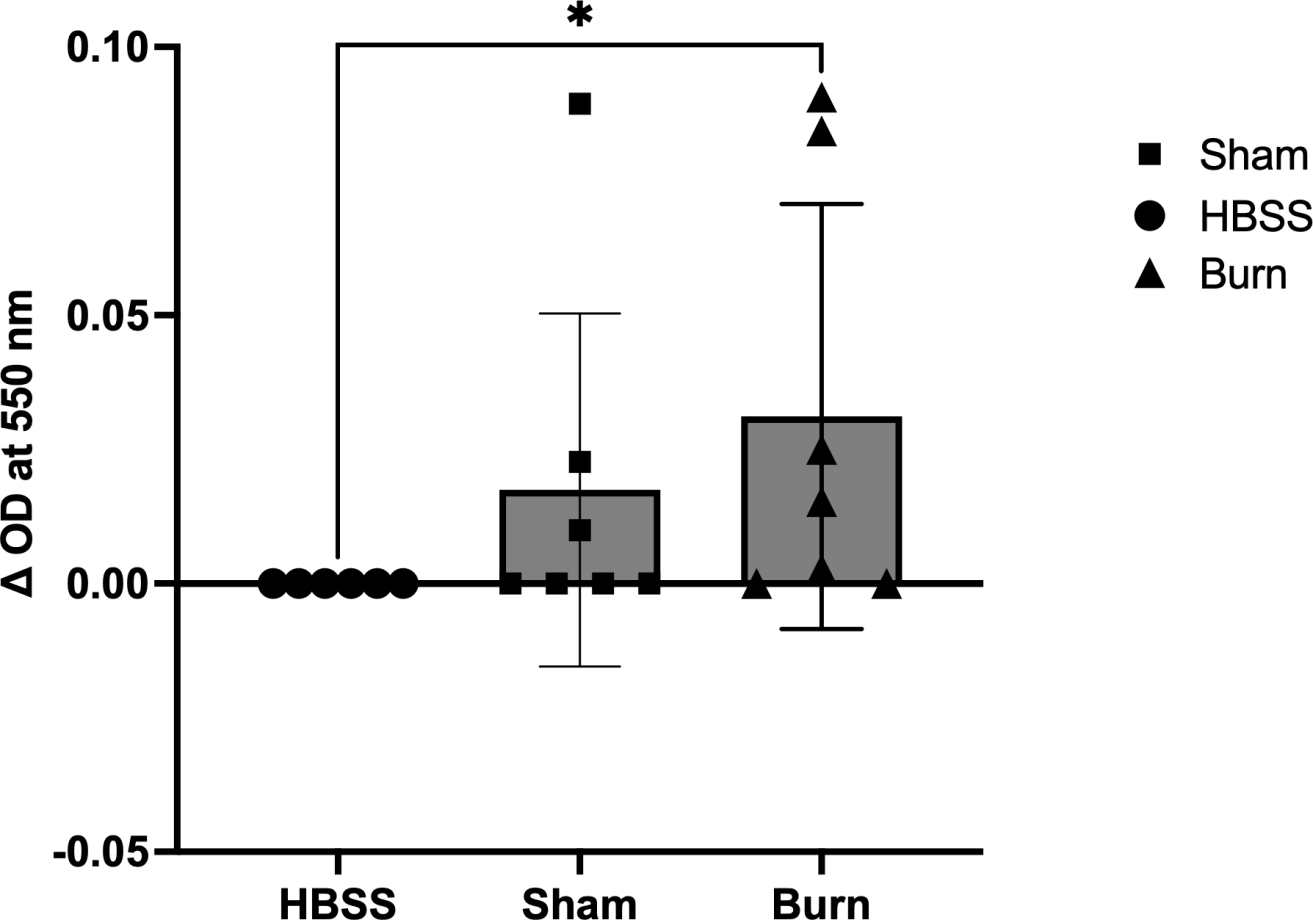
Resting neutrophils from Sham mice can be activated with sera from burned mice. Pooled neutrophils from Sham mice were incubated with HBSS^+^, sera from Sham mice, or sera from burned mice for 20 minutes at 37 °C. The neutrophils incubated with sera from burned mice were able to produce ROS and reduce cytochrome C. *P < 0.05.

Because the bacterial killing of leukocytes isolated from the circulation of burned mice was decreased, we wanted to determine whether the burn impacted ROS production. Using leukocytes isolated from Sham mice, we incubated the cells with cytochrome C in either HBSS^+^ or sera from Sham or burned mice. We found that incubation of leukocytes in the sera from burned mice was able to cause the production of ROS without the addition of the agonist PMA (Fig. 5). This was not the case when leukocytes were incubated in the sera of Sham mice.

We previously reported that a non-lethal burn injury transiently releases HMGB1, a pro-inflammatory DAMP, into the circulation. However, during burn and infection, HMGB1 was released at higher levels and increased until death (17). We speculated that the HMGB1 released into the circulation of burned mice may have prematurely stimulated the leukocytes in the circulation, such that they may have reduced their bactericidal capacity before they entered the PA-infected seroma. Resting leukocytes from Sham mice were resuspended in either HBSS, sera from unburned mice (Sham), or sera from burned and infected mice (with quantified HMGB1), then incubated with cytochrome C without stimulation by PMA. These resting leukocytes were able to significantly produce ROS when incubated with sera from burned and infected mice, suggesting that leukocytes could be activated while still in circulation prior to arriving at the infected burn site.

## DISCUSSION

**Fig 6:**
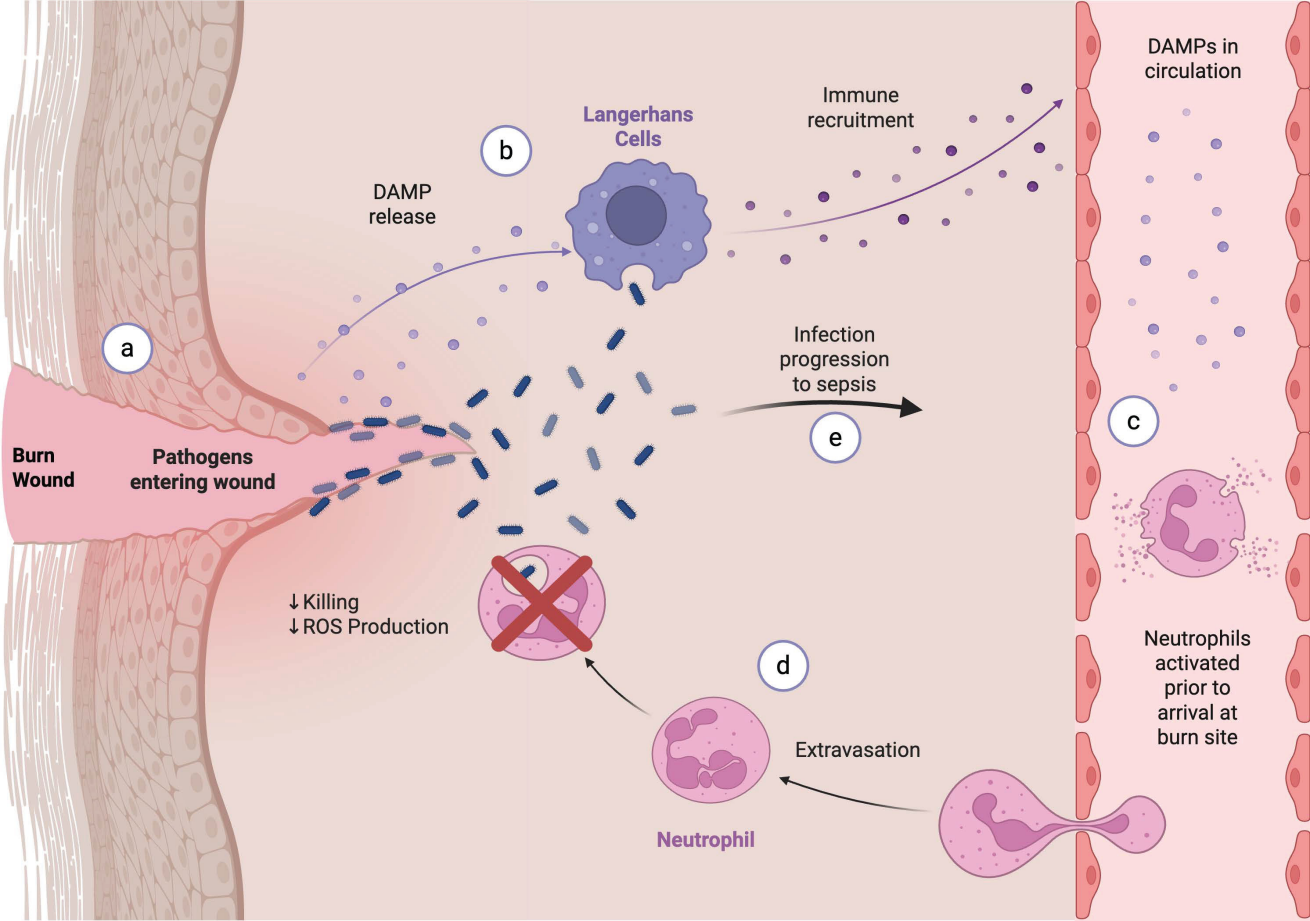
Proposed role of neutrophil dysfunction in burn wound sepsis. The skin barrier is broken, allowing for pathogens to enter the wound (**a**). DAMPs are also released from the damaged tissue, initiating an immune response (**b**). These DAMPs activate neutrophils while still in circulation (**c**). When the neutrophils are successfully recruited to the burn site, the burn environment further inhibits their activity, resulting in decreased ROS production and killing (**d**). These factors contribute to infection progression and increased mortality seen in our model (**e**) (29).

We compared the functional activity of leukocytes isolated from the circulation of Sham (not burned) mice, the circulation of burned mice, and the seroma of burned mice (Fig. 6) (29). We found that the PMNs from the circulation and seroma of burned mice were unable to kill PA in an opsonophagocytic assay compared to the PMNs from the circulation of Sham mice. The seroma fluid itself had a detrimental effect on PMN function. PMNs isolated from the circulation of both Sham and burned mice were unable to kill PA when the PMNs were resuspended in Sham fluid. This lack of bactericidal activity may have been due, in part, to the decreased ability of PMNs of the burned mice to generate ROS when compared to PMNs from Sham mice. When determining the effect of a non-lethal flame burn on ROS production, we found that the PMNs from the seroma of burned mice had impaired ROS activity following PMA stimulation when compared to the circulating PMNs isolated from the same mice. Finally, Sham PMNs incubated with sera from burned and from burned/infected mice produced ROS in the absence of stimulation, unlike the case when incubated with sera from Sham mice. Based on the data from flow cytometry, these PMNs in the B/I condition had less CD62L expression in the seroma at a key timepoint during infection, 12 hours. Together, this could indicate premature activation in combination with decreased function at the burn site, contributing to bacterial dissemination and mortality.

The ability of circulating PMNs from Sham mice incubated in the sera of burned mice to generate ROS in the absence of stimulation was correlated with the presence of HMGB1 in the sera of burned mice. We therefore speculated that the pro-inflammatory DAMP, HMGB1, released into the circulation from injured cells may have prematurely activated circulating PMNs and contributed to their dysfunction at the site of infection. The combination of the premature PMN activation as well as the inhibitory effect of seroma fluid on PMN function contributes to the marked susceptibility to lethal infection (4-log reduction in LD50) following burn injury.

There have been many studies of PMN function in patients and animal models following burn injury, often of >30% TBSA: leukocyte chemotaxis, degranulation, oxidative burst capacity, leukocyte extracellular trap (NET) formation, phagocytosis, apoptosis, and surface marker expression (30–32). Severe burn injury elicits a persistent inflammatory response with the release of pro-inflammatory mediators and release of immature leukocytes (33) and impaired bactericidal activity of PMNs (34). Serum levels of leukocyte elastase, myeloperoxidase, citrullinated histone H3, and complement factor C3a, as well as leukocyte counts, were increased on admission (35), suggesting that leukocyte activation and NETosis in burn injuries play a role in burn injury progression (35). Examination of oxidase activity of intact PMNs from patients with 12%–62% TBSA burns showed that superoxide anion generation in burn patients was decreased in burn patients compared to healthy controls (in part due to a deficiency of p47-phox and p67-phox components of the NADPH oxidase) (36). Single-cell transcriptome profiling of PMNs from burn patients showed that these burn patients, like our burned mice, had PMN activation (degranulation, chemotaxis, phagocytosis, and ROS production) that occurred early (day 1) and later (days 2 and 3) (37). Some observers reported infiltration of myeloid-derived suppressor cells that was not seen in the skin of non-burned mice (38). Miyazaki et al. observed in a murine model of non-lethal burn injury (20% TBSA) that ROS generation by PMN immediately after the burn caused pulmonary damage, but that ROS generation from the later (1–2 hours) ROS from the inflammatory response did not (39).

These studies document changes in leukocyte function during burn injury in both humans and rodents, but they did not correlate these defects with impairment in host defenses that facilitated infection as described in our studies. Furthermore, these models investigated severe and often fatal burns. Our model utilizes a 10% otherwise non-lethal burn, but still showed localized and disseminated effects on PMNs. The transient impairment in host defenses following burn injury suggests the need for early, aggressive therapeutic intervention during this period of greatest risk of lethal infection.

In a murine model of non-lethal (6% TBSA) burn injury similar to ours, Calum et al. reported a decrease in circulating PMNs with thermal injury (40). In our study, we attributed the leukopenia to the sequestration of cells within the seroma, which was not described in the Calum study. As was the case in our study, PA dissemination from the wound site was observed only in those mice with burns, not in Sham mice. They observed an increase in PMNs at the burn wound only in the PA-infected group, but the oxidative burst and phagocytic capacity of these PMNs were reduced. The increased local inflammatory response at the site of infection showed a reduced capacity to contain/eliminate the infection (40, 41) as evidenced by the distal dissemination of the PA from the burn wound. One potential explanation for the inability of PMNs to prevent dissemination in the Calum study may have been due to the premature activation of the PMNs by DAMPs before reaching the site of burn wound infection.

There is increasing recognition of the immunomodulatory role of DAMPs, such as HMGB1, that are released into the circulation from cells injured at the burn site. HMGB1 is a ubiquitous nuclear protein that maintains nucleosome integrity and facilitates gene transcription. These molecules are responsible in part for the pro-inflammatory response and subsequent tissue damage following thermal injury. Similar to endotoxin, which is also released into the circulation following thermal injury, HMGB1 activates the pro-inflammatory NF-κB signaling pathway through its binding to the TLR4 complex, resulting in the generation of pro-inflammatory cytokines. In our study, we compared the function of PMNs isolated from the circulation and burn site (seroma) of the same mice. While Hoiby reported that PMNs at the burn site of burned and infected mice had reduced oxidative burst and phagocytic capacity, we observed that the PMNs were dysfunctional even before they reached the burn site, which was correlated with elevated HMGB1 in the circulation following the burn.

In conclusion, our studies show that the increased susceptibility to lethal infection following burn injury is attributed in part to the premature activation of circulating PMNs before they reach the site of infection, as well as the inhibitory effect of seroma fluid on PMNs once they reach the site of infection. Therapies directed at HMGB1 or other DAMPs may improve the ability of burn patients to combat these life-threatening infections.
